# Adenomyoepithelioma of the breast with unusual confounding diagnostic feature: a case report

**DOI:** 10.1186/s13256-022-03507-3

**Published:** 2022-08-08

**Authors:** Liqa Al Mulla, Maha Abdelhadi, Afnan Al Muhanna, Tarek Elsharkawy, Areej Al Nemer

**Affiliations:** 1 Department of Pathology and Laboratory Medicine, Imam Abdulrahman Bin Faisal University–King Fahd Hospital of the University, King Faisal Road, Dammam, 34212 Saudi Arabia; 2 Breast Surgery Section, Department of General Surgery, King Fahd Hospital of the University, Al Khobar, Saudi Arabia; 3 Department of Radiology, King Fahd Hospital of the University, Al Khobar, Saudi Arabia

**Keywords:** Adenomyoepithelioma, Mucoepidermoid, Metaplasia, Myoepithelial

## Abstract

**Background:**

Adenomyoepithelioma of the breast is an uncommon subtype of breast neoplasm that occurs in adults over a wide age range but most commonly in middle-aged and older adults. It usually presents as a solitary palpable mass or is detected on breast radiographic images. Histologically, it is a biphasic tumor with proliferation of both the epithelial and myoepithelial components of the glands, with variable types of tissue metaplasia.

**Case presentation:**

A 64-year-old Saudi woman who underwent regular breast screening (mammogram) presented to our hospital following radiographic detection of a suspicious grouped microcalcification in the upper outer quadrant of her right breast on the mammogram. A wide local excision of the right breast lump was performed. Following histopathological examination of the breast lump, the final diagnosis was breast adenomyoepithelioma with mucoepidermoid/divergent differentiation, with no evidence of malignancy. About two years after the operation, a clinical follow-up conducted outside our hospital showed the development of ductal carcinoma *in situ* in the same breast.

**Conclusion:**

Although the prognosis and the plan of treatment remains the same, our case highlights the complexities in making an accurate diagnosis between the various types of metaplasia within adenomyoepithelioma on one hand and the presence of mucoepidermoid differentiation in adenomyoepithelioma on the other.

## Introduction

Adenomyoepithelioma (AME) is an uncommon breast neoplasm that was first described by Hamprel in 1970 [1]. The 2019 World Health Organization (WHO) classification of breast tumors defines adenomyoepitheliomas as biphasic neoplasms (usually) benign, characterized by small epithelium-lined spaces with inner luminal ductal cells and a proliferation of variably enlarged and clearly noticeable abluminal myoepithelial cells [2]. Here, we report a case of adenomyoepithelioma in the right breast of a woman with accompanying mucoepidermoid differentiation based on histological findings, an extremely rare occurrence.

## Case report

**Fig. 1:**
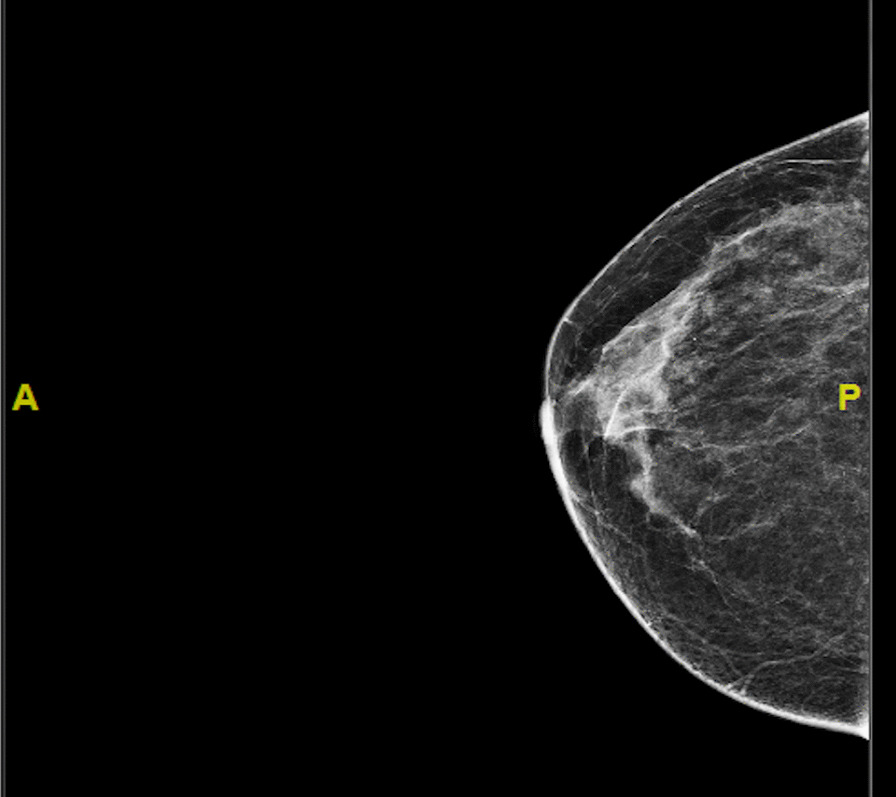
The breast mammogram (cranio-caudal view) showing an interval development of a suspicious grouped microcalcification in the upper outer quadrant of the right breast, Breast Imaging-Reporting and Data System 4C. **A** Anterior, **P** Posterior

**Fig. 2:**
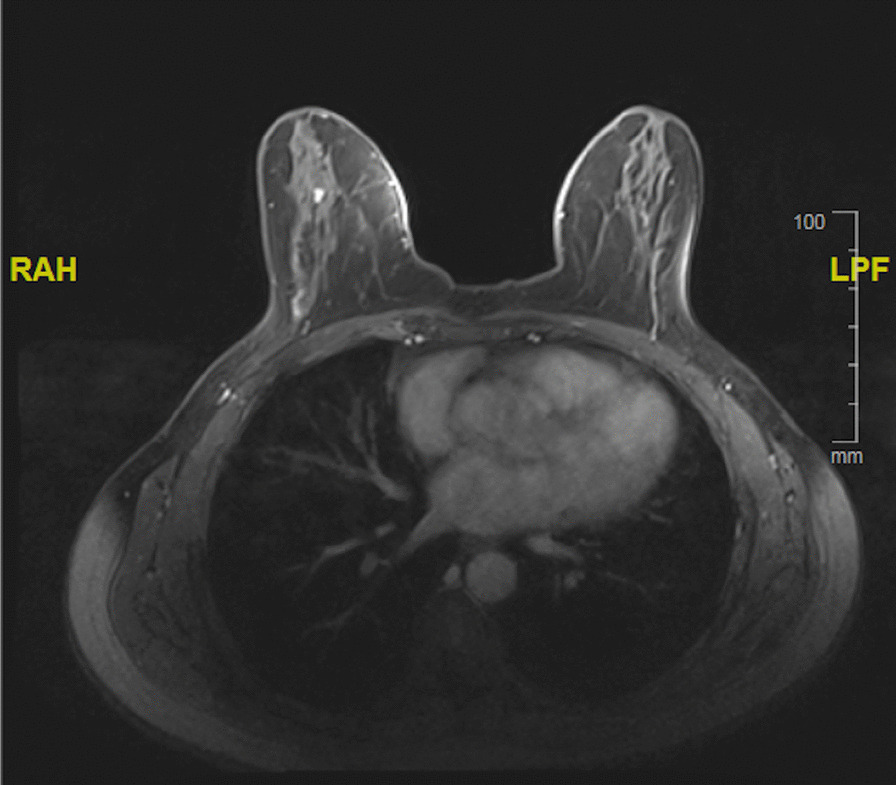
Breast magnetic resonance imaging showing non mass-like clumped progressive enhancement of microcalcification in the right breast. **RAH** Right, **LPF** Left

A 64-year-old Saudi woman with known hypertension and dyslipidemia, who underwent regular breast screening (mammogram), presented to our hospital following the detection of an interval development of a suspicious grouped microcalcification in the upper outer quadrant of the right breast on the mammogram. Risk assessment placed the microcalcification at Breast Imaging-Reporting and Data System (BI-RADS) 4C (Fig. 1). She denied any history of palpable mass or changes to the nipples or skin. The results of the breast examination were within the normal range. A complementary breast ultrasonography (US) showed grouped microcalcification with no definite mass, BI-RADS 4C. Breast magnetic resonance imaging (MRI) showed non mass-like clumped progressive enhancement corresponding to the mammogram findings of grouped microcalcification, BI-RADS 4C (Fig. 2). US-guided core needle biopsy revealed focal adenosis and microcalcification, stromal fibrosis, with no malignancy. Due to the high suspicion of malignancy, the breast surgery team elected to excise the lesion and the patient underwent US-guided wire localization and wide local excision under general anesthesia.

**Fig. 3:**
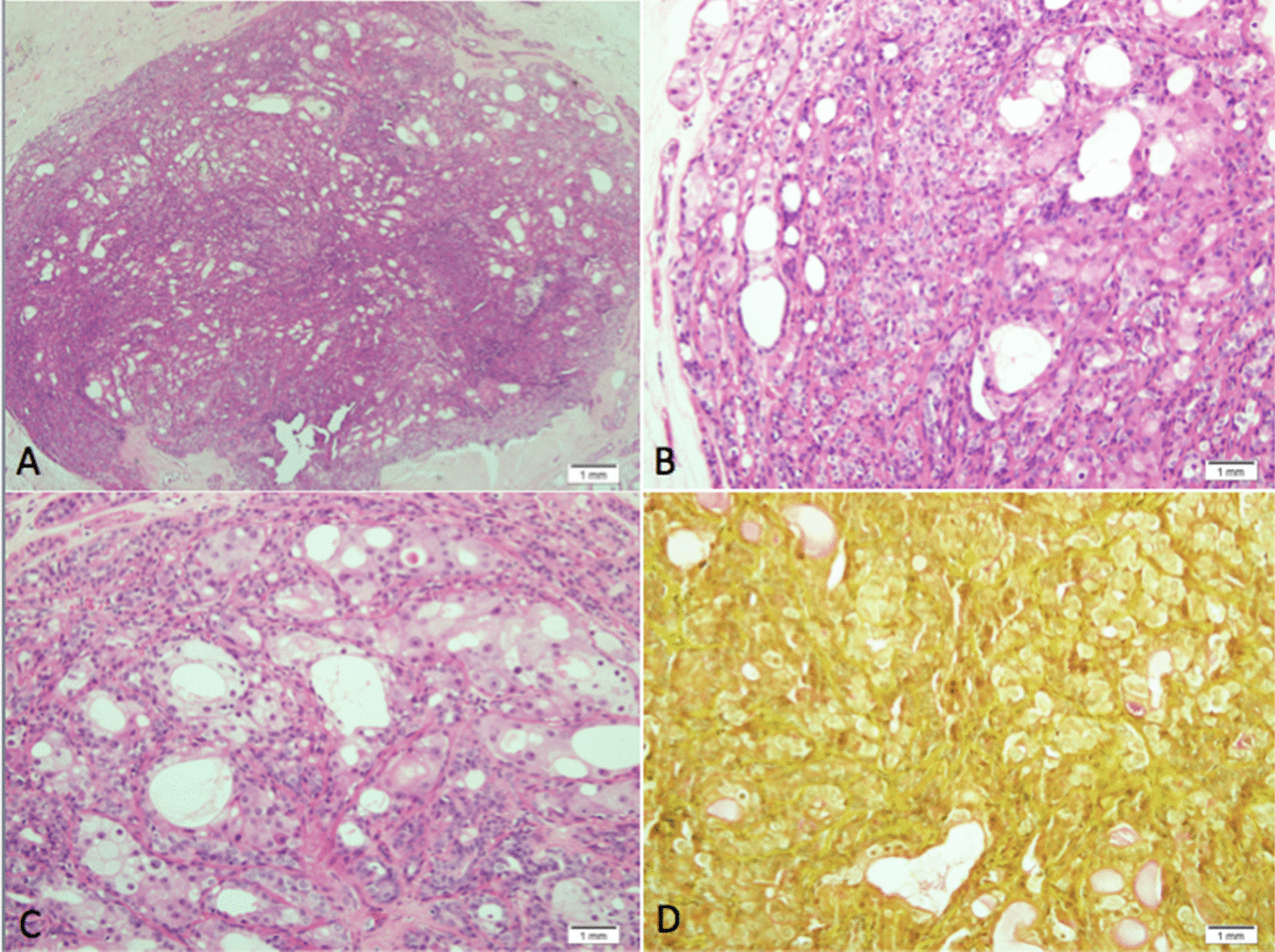
**a** Adenomyoepithelioma showing well-circumscribed nodule of proliferating glands (hematoxylin & eosin staining, magnification ×4). **b** Glands composed of bi-layered tubules with inner cuboidal luminal epithelial cells, and outer myoepithelial cells (hematoxylin & eosin stain, magnification ×200). **c** Sebaceous and apocrine metaplasia with occasional mucin-producing cells within the lesion (hematoxylin & eosin stain, magnification ×200). **d** Mucicarmine special stain demonstrates positive reactivity in the cytoplasmic mucin (mucicarmine special stain, magnification ×200)

The specimen was sent to the pathology lab as a breast lump with wire-guided localization, measuring 4.7 × 3 × 1 cm. The specimen was inked. Sectioning of the tissue revealed white-yellow homogenous cut surface with no definite masses or nodules. All of the tissue was formalin-fixed (10%) and embedded in paraffin for sectioning. Sections were stained with hematoxylin and eosin for histological examination along with immunohistochemistry and special stains. Microscopic study of the section revealed fibroadipose tissue with small-circumscribed nodule, about 0.7 cm in diameter (Fig. 3a), that was formed of proliferating epithelial and myoepithelial cells (Fig. 3b). Lobules of glands with tall-lining epithelium surrounded by myoepithelial cells were present. Areas of apocrine, squamous, and sebaceous metaplasia were present together with mucin-producing cells (Fig. 3c). Complementary immunohistochemistry studies revealed p63, calponin, and cytokeratin 5/6, demonstrating the positive reactivity of the myoepithelial component of the AME. Mucicarmine stain highlighted the cytoplasmic mucin (Fig. 3d). The pathological diagnosis was breast adenomyoepithelioma with mucoepidermoid/divergent differentiation, with no evidence of malignancy. The patient recovered uneventfully after the operation. A follow-up mammogram and US nine months after the procedure showed no evidence of recurrence. At about two years after the operation, a clinical follow-up conducted outside of our hospital noted the development of ductal carcinoma* in situ* in the same breast.

## Discussion

Breast adenomyoepithelioma is a rare neoplasm. It was reported for the first time in 1970 [1] and extensively classified by Tavassoli in 1991 [3]. Histopathology evaluation of specimens is highly required for the diagnosis of Adenomyoepithelioma due to the variable clinical presentation, imaging findings, and recent diagnostically challenging pathological features.

Clinically, AMEs usually occur in the fifth and sixth decades of life, are usually solitary, exceed one cm in size, and manifest as a well-circumscribed mass lesion [4–6]. Parikh *et al*. demonstrated various mammographic presentations of adenomyoepithelioma in their case series, ranging from masses to focal asymmetries, to microcalcifications [7]. In addition to the latest WHO definition of adenomyoepithelioma, Tavassoli used a mixture of architectural and cytological features to subdivid AME into three morphological patterns: lobulated, spindle cell, and tubular [3]. Most AMEs display the tubular pattern, typified by proliferation of luminal glandular cells rimmed by an outer layer of prominent myoepithelial cells with abundant clear cytoplasm [3, 6]. The myoepithelial cells in AME are more numerous and larger compared to cells of normal breast lobules, adenosis nodules, or simple papillomas. The presence of metaplastic changes within adenomyoepitheliomas has been reported in the literature. Notably, Young and Clement reported adenomyoepitheliomas with metaplastic apocrine cells and rare foci of squamous metaplasia, with cells replacing the columnar epithelial cells and occurring in the gland lumens [8]. Likewise, Tavassoli has described mucinous, apocrine, squamous, and sebaceous metaplasia [3], while Laforga *et al*. documented squamous and apocrine metaplasia [9]. It is not known if these metaplastic changes could affect the biological behavior of the tumor. The presence of such metaplastic changes reported within the AMEs should be differentiated from low-grade mucoepidermoid carcinoma (MEC).

Rosen has made the only report of a previously undescribed AME with mucoepidermoid differentiation, including microscopic images in his book [10]. In our case, we found the classic pathological features of AME along with apocrine, squamous and sebaceous metaplasia, and mucinous secretion in the glands, based on mucicarmine staining, which is in line with the diagnosis of adenomyoepithelioma with mucoepidermoid differentiation. All cell types are devoid of any malignant features.

Morphologically, AMEs are highly variable and their epithelial and myoepithelial components can present in a variety of patterns and metaplasias [7]. In the literature, there is a general agreement that all adenomyoepitheliomas should undergo wide excision with negative margins to reduce the risk of local recurrence and nodal metastases and to exclude malignancy [4, 11–14].

The breast is an uncommon location for MEC. Metaxa *et al*., in an extended review of the literature going back to the 1970s, noted that only 41 cases have been reported [15]. Cheng *et al*. reported the incidence of MEC in the breast to be 0.03%, but Fisher believes that it is 0.2–0.3% or even higher because these types of lesions may be misdiagnosed, such as atypical squamous metaplasia [16, 17]. Histologically, in the breast, MEC can be of low or high grade, similar to observations in the salivary glands. Low-grade MEC is characterized by the admixtures of various cells types, ranging from basaloid to mucinous to intermediate and to epidermoid, all organized in cystic and solid structures. High-grade MEC shows the same types of cells, but with a higher degree of atypia and usually arranged in solid structures [18].

Regarding the MEC, Patchefsky *et al*. were the first to present two cases of low-grade MEC of the breast [19]. Clinical features, therapeutic strategies, and the prognosis of MEC are related to its histological grading and the accuracy of existing literature. Those patients with low-grade tumors may be cured by complete resection as low-grade tumors are usually considered to be potentially curable, with a low risk of metastasis or recurrence [16].

## Conclusion

The diagnosis of adenomyoepithelioma with variable metaplasia on the one hand, and the existence of mucoepidermoid differentiation in adenomyoepithelioma on the other hand is truly confounding. The case described here illustrates the complexities in differentiating between the two and arriving at the accurate diagnosis, although prognosis and the treatment plan remain the same.

## Data Availability

The datasets used and/or analyzed to support the findings of this study are included within the article.

## Abbreviations

AME: Adenomyoepitheliomas
BI-RADS: Breast Imaging-Reporting and Data System
MEC: Mucoepidermoid carcinoma
MRI: Magnetic resonance imaging
US: Ultrasonography
WHO: World Health Organization
